# Unidirectional perfect absorber

**DOI:** 10.1038/srep32919

**Published:** 2016-09-12

**Authors:** L. Jin, P. Wang, Z. Song

**Affiliations:** 1 Nankai University, School of Physics, Tianjin, 300071, P.R. China

## Abstract

This study proposes a unidirectional perfect absorber (UPA), which we realized with a two-arm Aharonov-Bohm interferometer, that consists of a dissipative resonator side-coupled to a uniform resonator array. The UPA has reflection-less full absorption on one direction, and reflectionless full transmission on the other, with an appropriate magnetic flux and coupling, detuning, and loss of the side-coupled resonator. The magnetic flux controls the transmission, the left transmission is larger for magnetic flux less than one-half flux quantum; and the right transmission is larger for magnetic flux between one-half and one flux quantum. Besides, a perfect absorber (PA) can be realized based on the UPA, in which light waves from both sides, with arbitrary superposition of the ampli- tude and phase, are perfectly absorbed. The UPA is expected to be useful in the design of novel optical devices.

Symmetry and asymmetry are of considerable concern in fundamental physics and applications. In the past two decades, parity-time (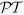) symmetry has been widely studied in non-Hermitian systems^1^,^2^,^3^,^4^,^5^,^6^,^7^,^8^,^9^,^10^,^11^,^12^,^13^,^14^,^15^,^16^,^17^,^18^,^19^,^20^,^21^,^22^,^23^,^24^, specifically, optical structures with a balanced gain and loss^25^,^26^,^27^,^28^,^29^,^30^,^31^,^32^,^33^,^34^,^35^,^36^,^37^,^38^,^39^,^40^. 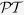 symmetry breaking was first observed in passive and active 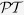-symmetric coupled waveguides^32^,^33^, and then in coupled microcavities^34^,^35^. In 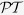-symmetric coupled microcavities, the transmission is reciprocal in the linear region, whereas nonreciprocal transmission caused by a gain-induced large nonlinearity occurs in the broken 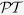-symmetric phase, which was realized with a novel on-chip optical isolator^34^,^35^. Light propagations are interest in 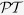 optical metamaterials, and unidirectional invisible and reflectionless transports have been demonstrated near the 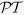-symmetric breaking point^36^,^37^. The reflection from one side is significantly suppressed, however, the transmissions remain symmetric. A coherent perfect absorber (CPA) yields a full absorption of coherently incident beams at a proper amplitude and phase from both sides without reflection. The CPA as a time-reversed laser, realizes an absorption process that is inverse of a laser emission; for incoherently input light fields, the absorption is reduced^38^,^39^,^40^; a 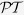-symmetric structure with a balanced gain and loss can simultaneously act as a laser and a CPA (known as the 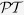-symmetric laser absorber), thereby supporting gain-induced lasing and loss-induced absorption^41^,^42^. These phenomena occur at spectral singularities of a non-Hermitian scattering system^43^,^44^. Unidirectional spectral singularities have recently been demonstrated by introducing Fano resonance in a coupled resonator array embedded a 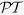-symmetric dimer with a balanced gain and loss. This system simultaneously supports unidirectional lasing and reflectionless absorption with finite reciprocal transmission^45^.

Reciprocal transmissions are found in 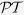-symmetric optical structures^36^,^37^, in which the 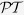 symmetry protects these reciprocal (symmetric) transmissions^46^,^47^,^48^,^49^,^50^. By applying the Aharonov-Bohm (AB) effect in dissipative optical systems, nonreciprocal (asymmetric) transmissions have been proposed^51^,^52^, where the magnetic field breaks the time-reversal symmetry and the reciprocality of light propagation. This method differs from previously designed asymmetric wave propagation techniques involving the use of nonlinearity^53^. The AB effect concerns a quantum phenomenon involving a charged particle affected by an electromagnetic field^54^,^55^. Although photons as neutral particles do not interact directly with magnetic fields, recent advances have led to the realization of effective magnetic fields for photons. The AB effect has recently been proposed and verified in optical systems through the magneto-optical effect^56^, dynamical modulation^57^, photon-phonon interactions^58^, and optical path imbalance^59^. Two special cases of unidirectional perfect absorber (UPA) with reflectionless full absorption on one side and reflectionless full transmission on the other was proposed^51^,^52^.

Here, this study details the basic conditions for realizing the UPA, specifically by a two-arm AB interferometer controlled through a synthetic magnetic flux. The AB interferometer consists of a dissipative resonator side-coupled to an array of coupled resonators. The side-coupled resonator provides another path for photons to tunnel through along the coupled resonator array. Photons from different pathways interact with each other, and therefore, the parameters of the side-coupled resonator, in addition to the enclosed magnetic flux, influence reflection and transmission. The magnetic flux controls the transmission, the left transmission is larger than the right transmission for magnetic flux less than one-half flux quantum; and the right transmission is larger than the left transmission for magnetic flux between one-half and one flux quantum. We show that the UPA can be realized with appropriate coupling, detuning, loss values, and the magnetic flux of the two-arm AB interferometer. Based on the properties of the UPA, we demonstrate a perfect absorber (PA), in which incident beams from both sides with arbitrary supposition of the amplitude and phase can be fully absorbed without reflection.

## Results

### Unidirectional perfect absorber

**Figure 1:**
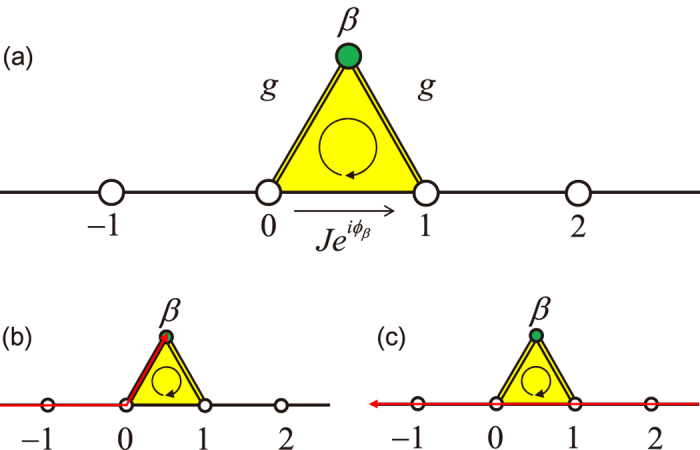
Unidirectional perfect absorber. (**a**) Schematic illustration of a two-arm Aharonov-Bohm (AB) interferometer of a side-coupled dissipative resonator (green site) that is side-coupled to a uniform resonator array, (**b**) left-input perfect absorption, (**c**) right-input perfect transmission.

A two-arm AB interferometer such as the one used in this study is shown in Fig. 1a. The two-arm AB interferometer consists of a dissipative (lossy) resonator and a passive resonator array. The passive resonators, with frequency *ω*_c_, are uniformly coupled; the coupling strength between the adjacent resonators is *J*. In the middle of the resonator array, a dissipative resonator is side-coupled to two adjacent passive resonators, the coupling strengths of which are both *g*. The dissipative resonator has a frequency *ω*_c_ + Δ and a loss Γ. An auxiliary resonator between resonator 0 and 1 as a mediate resonator introduces the phase imbalance of the photons that tunnelling through in different directions. Thus, the auxiliary resonator induces a nonreciprocal effective coupling between resonators 0 and 1, which is expressed as 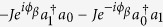. The effective magnetic flux is *ϕ*_*β*_ = 2*π*Δ*x*_*β*_/*λ*^59^, where 4Δ*x*_*β*_ represents the optical path length imbalance of the forward and backward directions in the auxiliary resonator between resonators 0 and 1, and *λ* denotes the resonant wavelength. Photons circling around the closed circle (0, 1, and *β*) feel an additional phase factor 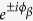 for the forward and backward directions.

Coupled mode theory details the dynamics of the coupled resonators^60^, and the equations of motion for resonators *j* < 0 and *j* > 1 are expressed as


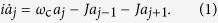


For resonators *j* = 0, *β*, and 1 in the closed circle, the equations of motion are expressed as


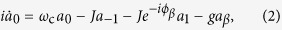



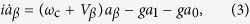



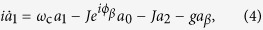


where the mode is 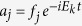, and the dispersion relation supported by the resonator array is *E*_*k*_ = *ω*_c_ − 2*J* cos *k*. The ring resonator supports two normal modes. We considered the clockwise mode without loss of generality. The magnetic flux introduced in the AB interferometer for counter-clockwise mode is obviously opposite that for clockwise mode (For any magnetic flux Φ for clockwise mode, the magnetic flux is −Φ for counter clockwise mode, and vise versa). With the following equation, we show that with an appropriate magnetic flux and side-coupled resonator coupling, detuning, and loss values, the AB interferometer displays reflectionless full absorption in one direction and full transmission in the other,


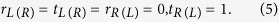


where the scattering matrix (*S* matrix) becomes a 2 × 2 Jordan block with two coalesced eigenvalues of zero.

In the side-coupled structure, photons can tunnel directly from the input to the output connection resonator; or they can first tunnel from the input resonator to the side-coupled resonator (*β*), and in turn to the output resonator. The two paths form a closed circle with an enclosed synthetic magnetic flux. The magnetic flux, and combined with the loss of the side-coupled resonator, destroys the time-reversal symmetry of tunneling, breaks the reciprocity of the transmission and alters the scattering coefficients. For the right (left) incident wave with a reflectionless full transmission, wave interference at the side-coupled resonator is completely destructive, and the side-coupled resonator is decoupled from the resonator array, and the loss does not play a role. However, when considering an incident wave from the opposite direction of the left (right) side, a destructive interference is require at output resonator 1 (0) for the AB interferometer to act as a UPA; and the side-coupled lossy resonator must fully absorb the incident beam. The UPA is realized with the appropriate magnetic flux, coupling, detuning, and loss of the side-coupled resonator.

For a UPA fully absorbing the left incident beam, the modal amplitudes satisfy *f*_*j*_ = *e*^*ikj*^ (*j* ≤ 0), and *f*_*j*_ = 0 (*j* > 0) for a left-input plane wave *e*^*ikj*^ with wave vector *k* as shown schematically in Fig. 1b. The completely destructive interference at resonator 1 (*f*_1_ = 0) and the dispersion relation (*E*_*k*_ = *ω*_c_ − 2 cos *k*) lead to *f*_*β*_ = (*J*/*g*)*e*^*ik*^. From the equation of motion for resonator 1, we obtain 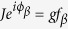, where the magnetic flux must be fixed at *ϕ*_*β*_ = *π* + *k*. The resonator detuning and loss values are obtained by solving Eq. (3), which yields *V*_*β*_ = (*g*^2^/*J*)*e*^−*ik*^ − 2*J* cos *k*. The interferometer parameters for the perfect absorption of the left incident wave is determined as follows. The magnetic flux satisfies


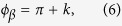


the detuning of the side-coupled resonator satisfies


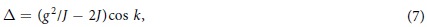


and the loss satisfies


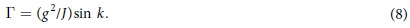


Under the conditions of Eqs (6, 7, 8), for a right-input *e*^−*ikj*^ (*j* > 0), the side-coupled resonator *β* is equivalently decoupled from the resonator array, and the modal amplitudes satisfy *f*_*j*_ = *e*^−*ikj*^ (*j* > 0), 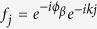 (*j*  0) and *f*_*β*_ = 0 (Fig. 1c). This corresponds to a reflectionless full transmission.

Under the condition a UPA fully absorbing the left incident beam, the synthetic flux induces an additional phase factor 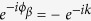 after the photons tunnel from resonator 1 to 0 for a right-input *e*^−*ikj*^ (*j* > 0), whereas the photons from resonators 1 (*e*^−*ik*^) and 0 (−*e*^−*ik*^) would cause destructive interference at resonator *β*. Thus, the right incident wave induces an equivalent isolation of side-coupled resonator *β*, and the right incident wave is fully transmitted. However, for left-input *e*^*ikj*^, because of the detuning and loss of the side-coupled resonator, we have *E*_*k*_ − *V*_*β*_ = −(*g*^2^/*J*)*e*^−*ik*^, hence we have the amplitudes *f*_0_ = 1, *f*_*β*_ = (*J*/*g*)*e*^*ik*^, and *f*_1_ = 0 at resonators 0, *β*, and 1. The left incident wave yields *r*_L_ = *t*_L_ = 0, which implies reflectionless full absorption.

For a UPA fully absorbing the right incident beam, the amplitude satisfies *f*_*j*_ = *e*^*ikj*^ (*j* ≥ 1) and *f*_*j*_ = 0 (*j* < 1) for the right-input plane wave *e*^−*ikj*^ with wave vector *k*. The completely destructive interference at resonator 0 and the dispersion relation lead to *f*_*β*_ = *J*/*g*. From the equation of motion for resonator 0, we obtain 
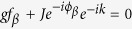
, for which the magnetic flux must be *ϕ*_*β*_ = *π* − *k*. Resonator detuning and loss are obtained in a similar manner from Eq. (3), *V*_*β*_ = (*g*^2^/*J*)*e*^−*ik*^ − 2*J* cos *k*, which yields the detuning and loss of the side-coupled resonator, as shown in Eqs (7 and 8). The side-coupled resonator *β* is equivalently decoupled from the resonator array for a left-input *e*^*ikj*^ (*j* < 1), and the modal amplitudes satisfy *f*_*j*_ = *e*^*ikj*^ (*j*  0), 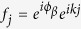 (*j* > 0) and *f*_*β*_ = 0, which correspond to a reflectionless full transmission.

In realizing the UPA, the detuning and loss of side-coupled resonator *β* are (*g*^2^/*J* − 2*J*)cos *k* and (*g*^2^/*J*)sin *k*, respectively. The magnetic flux controls the absorption and transmission of a UPA. When the magnetic flux is *ϕ*_*β*_ = *π* + *k*, we have a full absorption of the left input and a full transmission of the right input. Conversely, when the magnetic flux is *ϕ*_*β*_ = *π* − *k*, we have a full absorption of the right input, and a full transmission of the left input. We refer to these as two UPA types, in the same manner that we differentiate between two types of chiralities.

**Figure 2:**
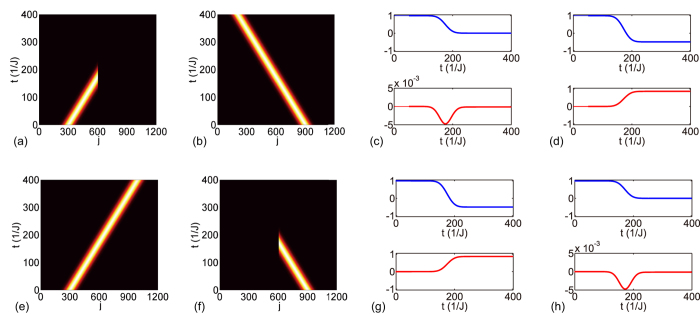
Perfect absorption and full transmission in opposite directions. Contour plots for the full absorption of the left input and full transmission of the right input in (**a,b**), and the full absorption of the right input and full transmission of the left input in (**e,f**) of a Gaussian profile |Ψ(*t*)〉. (**c,d,g,h**) Real (imaginary) part of overlap 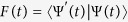 in the upper (lower) level in blue (red). The overlap is initially 1 before becoming 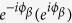 after passing the unidirectional perfect absorber (UPA) in the upper (lower) panel. |Ψ^′^(*t*)〉 represents the dynamics of the same Gaussian wave on the uniform array without the side-coupled resonator. The input wave vector is *k* = *π*/3, and the UPA parameters are 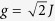, Δ = 0, 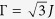, and *ϕ*_*β*_ = 4*π*/3 (*ϕ*_*β*_ = 2*π*/3) for **a–d** (**e–h**).

We numerically verified the reflection and transmission of a UPA. Considering a Gaussian wave


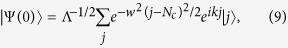


where *N*_c_ denotes the centre, *w* reflects the wave packet width in real space, *k* represents the velocity 2*J* sin *k* of the wave packet in the uniform resonator array, and Λ denotes a normalization constant. In the simulation, we considered two leads with finite sites *N* = 600 connected to the centre of the two-arm AB interferometer. The Gaussian wave has *w* = 0.02 and *k* = *π*/3. The side-coupled resonator has the parameters of detuning at Δ = 0 and loss at 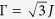, and is coupled to the resonator array at strength 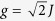. The magnetic flux is *ϕ*_*β*_ = 4*π*/3. Figure 2a,b show the contour plots of |Ψ(*t*)|^2^, which represents the time evolution of the Gaussian wave 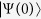 operating based on coupled mode theory. As shown in Fig. 2a, the Gaussian wave is initially centred at *N*_c_ = 300 with velocity 2*J* sin(*π*/3) ≈ 1.73*J* injected from the left and moving toward the right. The wave packet centre reaches the UPA at time 300/[2*J* sin(*π*/3)] ≈ 173(1/*J*), after which it is absorbed completely without reflection. As shown in Fig. 2b, the Gaussian wave is initially centred at *N*_c_ = 900, with velocity 1.73*J* from the right and moving toward the left. The wave packet reaches the UPA at time 173(1/*J*), after which it is transmitted completely without reflection. To compare the full transmission in the UPA with that in a uniform resonator array without side coupling, we calculated the time evolution of the same Gaussian wave packet in a uniform resonator array, denoted as 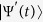. Figure 2c,d show the overlap 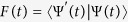 between the Gaussian wave in the UPA and in the uniform resonator array; the real part of *F*(*t*) is plotted in the upper panel in blue, whereas the imaginary part is plotted in the lower panel in red. After light waves pass through the AB interferometer, the overlap is 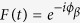. This indicates that the side-coupled lossy resonator is completely decoupled from the resonator array in the UPA. For the right incident wave, the wave packet dynamics in the UPA is identical to that in a uniform resonator array, except for an additional phase factor 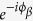 that results from the synthetic magnetic flux after wave pass through the UPA. Conversely, for the left incident wave, the wave packet is fully absorbed without reflection. The UPA has a unidirectional transmission, acting as a perfect diode. The reflections from both sides are zero. The magnetic flux and loss break the reciprocity of the transmission, but not that of reflection. The dynamics of a Gaussian wave packet are shown in Fig. 2e–h under the following condition: the magnetic flux is *ϕ*_*β*_ = 2*π*/3, where the right incident beam is fully absorbed, and left incident beam is fully transmitted. The overlap for left incident wave is 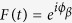, which indicates an additional phase factor 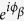 after wave pass through the UPA from the left side to the right side. Based on the unidirectional property of the UPA, we propose an PA with zero reflection and transmission from both sides as follows.

The UPA is a perfect optical diode, incident light is only one-way pass through. In one direction, light travelling is unaffected; but in the opposite direction, light travelling is forbidden and the incident light is fully absorbed. As an optical device, the UPA can be used to control light transport. For example, embedded a UPA that absorbing right incident light in the left side of a scattering centre, the scattering of a right incidence is reflectionless transmitted with transmission coefficient unchanged. Besides, the scattering of a left incidence is transmissionless reflected with reflection coefficient unchanged. The UPA rectifies light waves, the left-going waves after scattering vanish but the right-going waves are unaffected. The rectified light waves are right-going only. The magnetic flux *ϕ*_*β*_ is introduced through the optical path imbalance method^59^. The prime resonators 0 and 1 are evanescently coupled via an auxiliary resonator between them. The auxiliary resonator is the mediate resonator that connecting the prime resonators. The clockwise mode in the prime resonators turns to the counterclockwise mode in the auxiliary resonator when photons tunnelling from the prime resonators to the auxiliary resonator and vice versa. The perimeter of the prime resonators is integer resonant wavelength. The auxiliary resonator is antiresonant with the prime resonators 0 and 1, when the perimeter of the auxiliary resonator has an extra length of 3*λ*/2 than that of the prime resonators, the effective coupling strength between resonators 0 and 1 is −*J*. When the perimeter of the auxiliary resonator has an extra length *λ*/2 instead, the effective coupling strength induced is *J*, which equivalently creates a half-flux quantum effective magnetic flux in the closed circle *β*. A nonreciprocal phase factor can be introduced in the effective coupling through the optical path length difference of the auxiliary resonator. Photons travelling in the forward and backward directions acquire additional phase factors 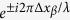. Thus, the effective magnetic flux 2*π*Δ*x*_*β*_/*λ* introduced is proportional to the difference of the two optical paths. Photons travelling in the forward direction (tunnelling from resonator 0 to resonator 1) experience an extra 4Δ*x*_*β*_ length than travelling in the backward direction (tunneling from resonator 1 to resonator 0). Through tuning the auxiliary resonator position in the coupling process, the optical path lengths and their difference are changed accordingly. An optical length difference *λ* (i.e., Δ*x*_*β*_ = *λ*/4) results in a quarter quantum magnetic flux *π*/2. To realize effective magnetic flux *ϕ*_*β*_ = *π* ± *k*, the auxiliary resonator perimeter length is chosen *λ*/2 longer than the prime resonators and the optical path length difference in the coupling process should be 2*kλ*/*π* [i.e., Δ*x*_*β*_ = *kλ*/(2*π*)].

A two-arm AB interferometer with a side-coupled dissipative resonator typically involves a reciprocal reflection |*r*_L_| = |*r*_R_|; however, the transmission is nonreciprocal in general case. We define an asymmetric transmission contrast to characterize quantitatively the unidirectionality,


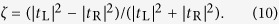


For a passive side-coupled resonator *γ* = *mπ*, or a trivial magnetic flux *ϕ*_*β*_ = *nπ* (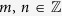), the transmission is reciprocal and we have *ζ* = 0. However, for a dissipative/active side-coupled resonator *γ* ≠ *mπ*, the transmission is unidirectional for nontrivial magnetic flux *ϕ*_*β*_ ≠ *nπ* (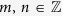). As shown in the Appendix, the transmission satisfies |*t*_L_(*ϕ*_*β*_)| = |*t*_R_(−*ϕ*_*β*_)|, therefore, the asymmetric transmission contrast satisfies *ζ*(−*ϕ*_*β*_) = −*ζ*(*ϕ*_*β*_). At unidirectional transmission zeros, i.e., *t*_L(R)_ = 0, *t*_R(L)_ ≠ 0, the contrast reaches *ζ* = −1(+1) for maximally unidirectional transmission. Consider a dissipative side-coupled resonator, the magnetic flux breaks the reciprocality of transmission. In a magnetic flux period of (0, 2*π*), the left transmission is larger than the right transmission in region 0 < *ϕ*_*β*_ < *π*; Conversely, when the magnetic flux is in region *π* < *ϕ*_*β*_ < 2*π*, the left transmission is smaller than the right transmission. When *V*_*β*_ = (*κ*^2^/*J*)*e*^−*iγ*^ − 2*J* cos *k*, the asymmetric transmission contrast *ζ* is independent of the incident wave vector *k*, and is expressed as


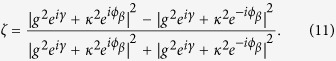


*ζ* depends on the magnetic flux *ϕ*_*β*_ as well as the coupling, detuning, and loss of the side-coupled resonator, as determined respectively by parameters *g*, *κ*, and *γ*.

**Figure 3:**
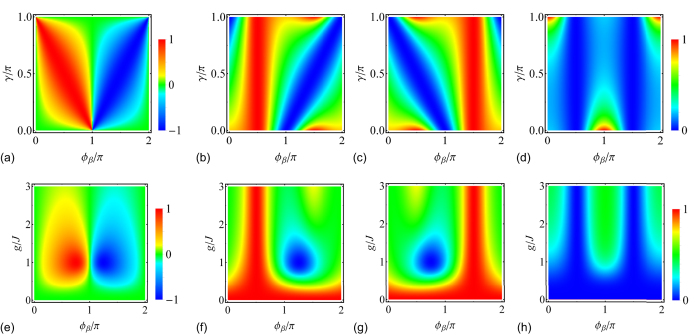
Transmission contrast and scattering probability. (**a**–**d**) for *κ* = *g*. (**e**–**h**) for *κ* = *J*. (**a,e**) Contour plot of the asymmetric transmission contrast *ζ*. In (**a**), the parameter of the side-coupled resonator is *V*_*β*_ = (*g*^2^/*J*)*e*^−*iγ*^ − 2*J* cos *k*, *ζ* is independent of the incident wave vector and coupling *g*. Transmission zeros are at *ϕ*_*β*_ = *π* ± *γ*; in (**e**), the parameter of the side-coupled resonator is *V*_*β*_ = *Je*^−*iγ*^ − 2*J* cos *k*, *γ* = *π*/4. The transmission zeros are at *g*/*J* = 1, *ϕ*_*β*_ = 3*π*/4, 5*π*/4. Contour plots of (**b,f**) left transmission |*t*_L_|^2^, (**c,g**) right transmission |*t*_R_|^2^, and (**d,h**) left/right reflection |*r*_L/R_|^2^ for incident wave vector *k* = *π*/2. The color bars for (**b**–**d**) and (**f**–**h**) are shown in (**d**,**h**).

We plot the asymmetric transmission contrast *ζ* (Fig. 3a) for *κ*/*g* = 1. Under this condition, the left and right reflections and transmissions are all independent of coupling strength *g*. At *ϕ*_*β*_ = *π* + *γ*, the left transmission is *t*_L_ = 0. Conversely, at *ϕ*_*β*_ = *π* − *γ*, the right transmission is *t*_R_ = 0. Figure 3b,c show the unidirectional transmission for the left and right incident beams with *k* = *π*/2. The left transmission (Fig. 3b) is near unity at magnetic flux *ϕ*_*β*_ = *π*/2, whereas the right transmission (Fig. 3c) is near unity at magnetic flux *ϕ*_*β*_ = 3*π*/2. Figure 3d shows the reciprocal reflection for incident beam with *k* = *π*/2. The reflection is small, being less than 0.25 in a large region, and approximately zero near *ϕ*_*β*_ = *π*/2 and 3*π*/2.

Figure 3e shows the asymmetric transmission contrast *ζ* for *κ*/*J* = 1, and *γ* = *π*/4. Under this condition, the transmission zeros are at *g*/*J* = 1 when the magnetic flux is *ϕ*_*β*_ = 5*π*/4 for the left incident beam (*t*_L_ = 0), and when the magnetic flux is *ϕ*_*β*_ = 3*π*/4 for the right incident beam (*t*_R_ = 0). In Fig. 3f,g show the unidirectional transmissions for the left and right incident waves with vector *k* = *π*/2. The left transmission (Fig. 3f) is near unity at the magnetic flux *ϕ*_*β*_ = *π*/2, and near zero at the magnetic flux *ϕ*_*β*_ = 5*π*/4, *g*/*J* = 1. The right transmission (Fig. 3g) is near unity at the magnetic flux *ϕ*_*β*_ = 3*π*/2, and near zero at the magnetic flux *ϕ*_*β*_ = 3*π*/4, and *g*/*J* = 1. Figure 3h shows the reciprocal reflection for the incident wave with *k* = *π*/2; the reflection is less than 0.5 and approximately zero near *ϕ*_*β*_ = *π*/2, 3*π*/2.

For the input with wave vector *k*, at a magnetic flux of *ϕ*_*β*_ = *π* − *k* (*π* + *k*), e.g., *ϕ*_*β*_ = *π*/2 (3*π*/2) as shown in Fig. 3, the dissipative side-coupled resonator is isolated for the left (right) input, and we obtain |*r*_L(R)_|^2^ + |*t*_L(R)_|^2^ = 1; otherwise, the input is absorbed by the side-coupled resonator, and we obtain |*r*_L(R)_|^2^ + |*t*_L(R)_|^2^ < 1.

### Perfect absorber

**Figure 4:**
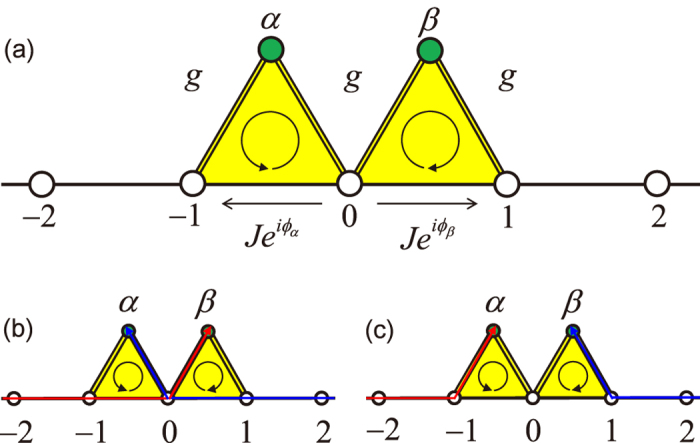
Perfect absorber. (**a**) Schematic illustration of a double two-arm AB interferometer of two dissipative resonators (green sites) side-coupled to a uniform resonator array. (**b**) PA at *ϕ*_*α*_ = *ϕ*_*β*_ = *π* + *k*. (**c**) PA at *ϕ*_*α*_ = *ϕ*_*β*_ = *π* − *k*.

Inspired by the UPA, we devised our proposed double two-arm AB interferometer, which can act as a PA when it is composed of a combination of two UPAs with different chiralities. A PA perfectly absorbs coherently and incoherently input light beams. As shown in Fig. 4a, the PA is a double two-arm AB interferometer enclosed two tunable magnetic fluxes. The double two-arm AB interferometer consists of two dissipative resonators side-coupled to a passive resonator array in the middle. The synthetic magnetic fluxes are introduced through auxiliary resonators^59^. The effective magnetic fluxes in the left (right) circles are *ϕ*_*α*(*β*)_ = 2*π*Δ*x*_*α*(*β*)_/*λ*, where Δ*x*_*α*(*β*)_ represents the optical path imbalance of the forward and backward directions between resonators 0 and −1 (1). Coupled mode theory details the dynamics^60^. The equations of motion for the resonators *j* < −1 and *j* > 1 are obtained as


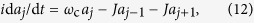


for resonators *j* = −1, *α*, *β*, 1, and 0, the equations of motion are obtained as


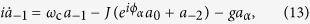



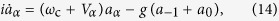



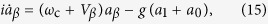



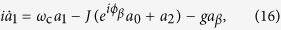



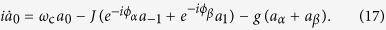


To calculate the coefficients, we employ the Jost solutions of modal amplitudes *f*_*j*_ = *Ae*^*ikj*^ + *Be*^−*ikj*^ for *j* < 0, and *f*_*j*_ = *Ce*^*ikj*^ + *De*^−*ikj*^ for *j* > 0^44^,^45^. The amplitudes for *j* = 0, *α*, and *β* are set to *f*_0_, *f*_*α*_, and *f*_*β*_, respectively. When the detunings and losses of the two side-coupled resonators satisfy *V*_*α*_ = *V*_*β*_ = (*g*^2^/*J*)*e*^−*ik*^ − 2*J* cos *k*, the equations of motion are simplified to


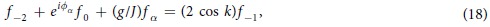



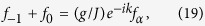



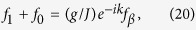



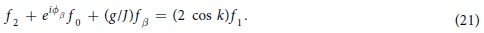



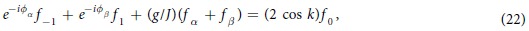


A UPA is recovered at magnetic fluxes *ϕ*_*α*_ = *π* + *k* and *ϕ*_*β*_ = *π* − *k* or *ϕ*_*α*_ = *π* − *k* and *ϕ*_*β*_ = *π* + *k*. In this study, the double two-arm AB interferometer is a combination of two UPAs with the same chirality. For instance, when *ϕ*_*α*_ = *π* + *k* and *ϕ*_*β*_ = *π* − *k*, the respective configurations of UPA-*α* and UPA-*β* both involve the left incident wave being fully transmitted while the right incident wave is fully absorbed. Hence, the left incident wave is fully transmitted with an additional phase factor 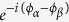 acquired. The right incident wave is to be absorbed at resonator *β*.

The double two-arm AB interferometer acts as an PA when it is combined with two UPAs with different chiralities at the magnetic fluxes *ϕ*_*α*_ = *ϕ*_*β*_ = *π* + *k* (i.e., the configuration of UPA-*α* (UPA-*β*) enables it to absorb the right (left) incident wave). From the equation of motion shown in Eq. (18, 19, 20, 21, 22), we obtain *B* = *C* = 0, and the amplitude at the centre resonator 0 satisfies *e*^*ik*^*f*_0_ = −*A* − *D*. The amplitudes at the side-coupled resonators satisfy *f*_*α*_ = −(*J*/*g*)*D* and *f*_*β*_ = −(*J*/*g*)*A*. The incident beam from left side is perfectly absorbed at resonator *β*, whereas that from the right side is perfectly absorbed at resonator *α*, as shown schematically in Fig. 4b. Conversely, when the magnetic fluxes are *ϕ*_*α*_ = *ϕ*_*β*_ = *π* − *k* (i.e., the configuration of UPA-*α* (UPA-*β*) enables it to absorb the left (right) incident wave), we have *B* = *C* = 0, *f*_*α*_ = (*J*/*g*)*A*, *f*_*β*_ = (*J*/*g*)*D*, and *f*_0_ = 0. Under this condition, the incident waves from both sides are perfectly absorbed with a destructive interference at resonator 0 as shown schematically in Fig. 4c.

Therefore, for side-coupled resonators with detunings Δ_*α*_ = Δ_*β*_ = (*g*^2^/*J* − 2*J*) cos *k* and losses Γ_*α*_ = Γ_*β*_ = (*g*^2^/*J*)sin *k*, when the magnetic fluxes are *ϕ*_*α*_ = *ϕ*_*β*_ = *π* ± *k*, an arbitrary superposition of the amplitude and phase for light incident from both sides are fully absorbed as long as the incident wave vectors are *k*. The reflection and transmission coefficients for the PA are given by


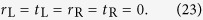


**Figure 5:**
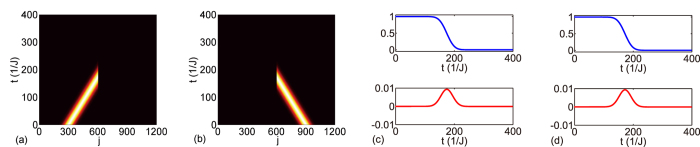
Perfect absorption of wave from either side. (**a,b**) Full absorption of a Gaussian wave |Ψ(*t*)〉. (**c,d**) Real (imaginary) part of overlap 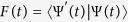 in the upper (lower) level in blue (red). |Ψ^′^(*t*)〉 is the dynamics of the same Gaussian wave on the uniform resonator array without side-coupled resonator. The input wave vector is *k* = *π*/3. The PA parameters are *ϕ*_*α*_ = *ϕ*_*β*_ = 4*π*/3, 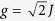, Δ = 0, and Γ = 2*J* sin *k*.

**Figure 6:**
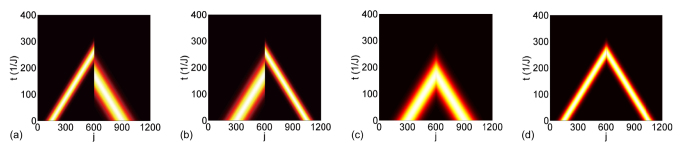
Perfect absorption of superposition waves from both sides. Perfect absorption of two Gaussian waves with different widths (*w* = 0.01 and 0.02), centred at different sites and input from different sides. The incident wave vector is *k* = *π*/3, the parameters of PA are *ϕ*_*α*_ = *ϕ*_*β*_ = 4*π*/3, 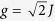, Δ = 0, and Γ = 2*J* sin *k*. In (**a,b**), the relative phase between the left wave packet and right wave packet is 0; in (**c**,**d**), the relative phase is *π*/2.

As shown in Fig. 5a,b, we performed the time evolution of a Gaussian wave packet input from the left and right sides, respectively. The Gaussian wave has *w* = 0.02 and its velocity is 2*J* sin(*π*/3). The side-coupling strength is 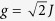, and the detuning is Δ = 0, the loss is 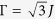, and the magnetic fluxes are *ϕ*_*α*_ = *ϕ*_*β*_ = 4*π*/3. The overlap between the Gaussian wave packet in a PA and that in a uniform resonator array is shown in Fig. 5c,d; the overlap is 1 before the wave packet reaches the PA, and decreases to 0 upon passing the PA. The incident wave from either side is fully absorbed without reflection. Figure 6 displays the perfect absorption of a superposition of two Gaussian waves incident from both sides. The Gaussian wave packets have the same vector *k* = *π*/3, a relative phase *e*^*iψ*^ is applied on the wavepacket in the right side as an overall phase. In Fig. 6a,b, the wave packets with different widths are centred asymmetrically about the PA, the relative phase applied is 0; in Fig. 6c,d, the wave packets with the same width are centred symmetrically about the PA, the relative phase applied is *ψ* = *π*/2. As shown in Fig. 6, two Gaussian wave packets are absorbed independently, which are attributed to the two UPAs that constructed the PA. Therefore, the perfect absorption does not require coherently input of light waves in the two sides but requires the wave vector matching the PA absorption frequency.

Perfect absorption is irrelevant to the distance of UPA-*α* and UPA-*β*. As shown in Fig. 4, two UPAs *α* and *β* with different chiralities are the nearest neighbors. For UPA-*α* and UPA-*β* being fully separated, perfect absorption remains. Under this condition, the incident beam fully passes the first UPA without reflection before the second UPA absorbs it perfectly, or it is perfectly absorbed by the first UPA encountered for the incident light comes from either direction. This is a consequence of the UPA simultaneously supporting perfect absorption and perfect transmission in opposite directions, respectively. However, when two UPAs coincide in position, two dissipative resonators becomes coupled to the same two adjacent resonators in the coupled resonator array. The PA structure breaks, and the system becomes a three-arm AB interferometer. In this configuration, through symmetric and anti-symmetric combinations of the two side-coupled lossy resonators, we equivalently obtain a two-arm structure (Fig. 1) with 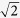 times the strength of side-coupling. Therefore, with appropriate detuning Δ_*α*_ = Δ_*β*_ = 2(*g*^2^/*J* − *J*)cos *k* combined with losses Γ_*α*_ = Γ_*β*_ = 2(*g*^2^/*J*)sin *k*, the UPA recovers from magnetic fluxes that are *ϕ*_*α*_ = *π* + *k* with *ϕ*_*β*_ = *π* − *k* or *ϕ*_*α*_ = *π* − *k* with *ϕ*_*β*_ = *π* + *k* in the three-arm AB interferometer.

The unidirectional perfect absorption is recovered in the double two-arm AB interferometer when it is combined by two UPAs with the same chirality. The light flow is controlled by the magnetic fluxes in the double two-arm AB interferometer, perfect absorption in one direction or in both directions is determined by the chirality of the two UPAs. Through changing the magnetic fluxes enclosed, the double two-arm AB interferometer can realize either side incidence being fully transmitted without reflection, either side incidence being fully absorbed, and both sides incidences being absorbed at appropriate resonators loss and incident wave vector. In the combination of UPAs with the same or different chirality, the dynamical properties of the perfect transmission in one side meanwhile perfect absorption in the other side with vanishing reflections play key roles in manipulating light propagation.

**Figure 7:**
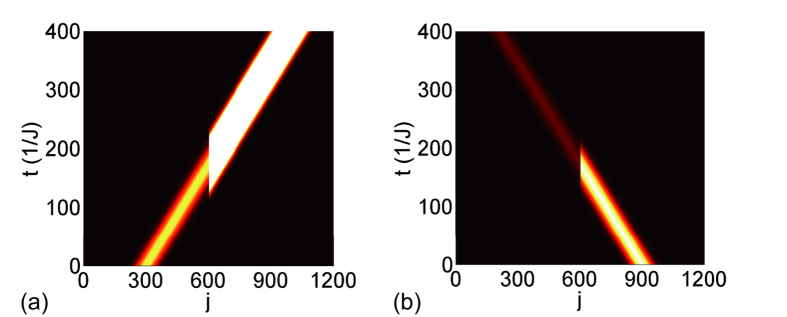
Unidirectional amplification and attenuation in opposite incident directions. Time evolution profile of a Gaussian wave for (**a**) left incidence and (**b**) right incidence. The system parameters are *V*_*α*_/*J* = 0.5*i*, *V*_*β*_/*J* = −0.5*i*, *ϕ*_*α*_ = *ϕ*_*β*_ = 2*π*/3, the wave vector is *k* = *π*/3.

In general case, at magnetic fluxes *ϕ*_*α*_ = *ϕ*_*β*_ = *π* ± *k*, the light scattering is both sides reflectionless. The magnetic fluxes 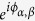 cause additional phase factors −*e*^ ± *ik*^ on the resonator couplings, which lead to a cancellation of the reflections *r*_L_ = *r*_R_ = 0. The transmission coefficients are related to the two side-coupled resonators. For side-coupled resonators being the same (different), the reflectionless transmission is symmetric (asymmetric). The side-coupled resonator with loss leads to the incident light wave attenuation. Thus, the symmetric (asymmetric) side-coupled resonators with losses induce directional (unidirectional) wave attenuation. On the contrary, the side-coupled resonator with gain results in the incident light wave amplification. Thus, a double two-arm AB interferometer can function differently. A structure having both side-coupled resonators with the same (different) loss or gain generates symmetric (asymmetric) attenuation or amplification. The loss is from the resonator dissipation, the gain is from the ion-doped active resonator under pump. In the case of a side-coupled structure with both gain and loss resonators, the incident light wave is amplified and attenuated in opposite propagating directions, the system functions as a unidirectional attenuator and amplifier. Figure 7 shows the dynamics of a Gaussian wave packet with *k* = *π*/3 input from opposite directions. The magnetic fluxes are set at *ϕ*_*α*_ = *ϕ*_*β*_ = 2*π*/3, this equivalently happens at the auxiliary resonators that connecting resonators −1, 0, 1 with additional perimeter lengths 3*λ*/2 and the path length differences in the connecting resonators from resonator 1 to 0 and from resonator 0 to −1 both being 2*λ*/3 (i.e., Δ*x*_*α*_ = Δ*x*_*β*_ = −*λ*/6). For the system simulated in Fig. 7, the side-coupled resonator frequencies are resonant with the resonator chain array, the gain and loss rates are Γ = *J*/2. The scattering is reflectionless (*r*_L,R_ = 0) with amplified left transmission |*t*_L_| ≈ 2.35 but attenuated right transmission |*t*_R_| ≈ 0.43.

## Conclusion

This study proposed a two-arm AB interferometer as a UPA, where light beams pass in a unidirectional manner. The AB interferometer consists of a side-coupled dissipative resonator, with an enclosed synthetic magnetic flux, which controls the light flow. The left transmission is larger than the right transmission for magnetic flux less than 1/2 flux quantum; and left transmission is smaller than right transmission for magnetic flux between 1/2 and 1 flux quantum. When the side coupling, detuning and loss of the coupled resonator are set to the appropriate values, the UPA allows reflectionless full absorption in one direction and reflectionless full transmission in the other. Based on the unidirectional property of the UPA, a PA is designed by combining two UPAs with different chiralities. The PA enables a perfect absorption of incident beams from both sides at arbitrary superpositions of the amplitude and phase. The magnetic flux is a useful source for the realization of fantastic scattering. The PA can also act as a UPA controlled by magnetic fluxes, perfect transmission or absorption in either side is possible. The magnetic flux, associated with non-Hermitian gain/loss plays a critical role in optical control, which is expected to be useful for the design of novel optical devices, such as optical diode, one-way controller, and reflectionless unidirectional amplifier and attenuator.

## Methods

### Scattering of the two-arm AB interferometer

The method presents the scattering coefficients of the two-arm AB interferometer shown in Fig. 1a. The resonator array is uniformly coupled at coupling strength *J*. The side-coupled resonator couples to the resonator array at coupling strength *g*, and for an incident beam with wave vector *k*, we consider the detuning and loss of the side-coupled resonator as *V*_*β*_ = *κ*^2^*e*^−*iγ*^ − 2*J* cos *k*, where the stationary resonator modal amplitudes are superposition of left- (*e*^*ikj*^) and right-going (*e*^−*ikj*^) plane waves,


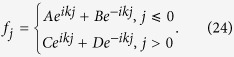


By setting *D* = 0 in *f*_*j*_, we obtain the scattering result for the left incident beam, and the left reflection coefficient is obtained from *r*_L_ = *B*/*A*, whereas the left transmission coefficient is obtained from *t*_L_ = *C*/*A*. Conversely, by setting *A* = 0 in *f*_*j*_, we obtain the scattering result for the right incident beam, the right reflection coefficient of which is obtained from *r*_R_ = *C*/*D*, whereas the right transmission coefficient is obtained from *t*_R_ = *B*/*D*.

### Scattering coefficients

The reflection and transmission coefficients are calculated from the equations of motion. Substituting Eq. (24) into Eqs (2, 3, 4), we obtain the scattering coefficients after simplification, which satisfy the relations |*t*_L_(*ϕ*_*β*_)| = |*t*_R_(−*ϕ*_*β*_)| and |*r*_L_| = |*t*_R_|, and the transmissions are


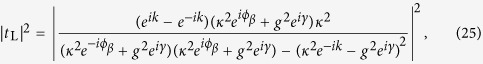



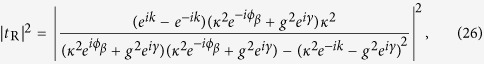


the reflections are


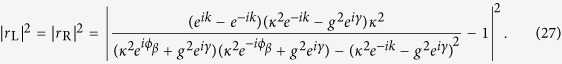


## Additional Information

**How to cite this article**: Jin, L. *et al*. Unidirectional perfect absorber. *Sci. Rep.* **6**, 32919; doi: 10.1038/srep32919 (2016).
